# Infiltrating myoepithelial carcinoma of the breast, a case report and cytologic-histologic correlation

**DOI:** 10.1186/1746-1596-3-7

**Published:** 2008-02-08

**Authors:** Daniel Lingamfelter, Yayan Chen, Kiyoe Kure, Kamani Lankachandra

**Affiliations:** 1 Department of Pathology, University of Missouri-Kansas City School of Medicine and Truman Medical Centers, Kansas City, Missouri, USA; 2 Department of Pathology, The Methodist Hospital, Houston, Texas, USA

## Abstract

**Introduction:**

Infiltrating myoepithelial carcinoma remains a rarely encountered lesion of the breast. The few cases that have surfaced firmly document the histopathology of this tumor, but its cytologic characteristics seemingly have been described in only one other report.

**Case presentation:**

Here we present the cytologic findings from a case of infiltrating myoepithelial carcinoma of the breast in a 52-year-old female and provide a histologic correlation with the subsequent biopsy and mastectomy specimens. While the cytology specimens displayed more myoepithelial cellular heterogeneity than was present on histology, a number of cytologic features including hypercellularity, pleomorphic spindle cells, and mitotic activity correlated well with the histopathology.

**Conclusion:**

The role of fine needle aspiration in the diagnosis of mammary myoepithelial carcinoma, in this case, was to establish malignancy rather than to arrive at a specific diagnosis, as a number of different entities potentially can mimic this neoplasm on cytologic specimens.

## Introduction

Myoepithelial carcinoma of the breast (MEC), a lesion composed purely of malignant myoepithelial cells, remains a rarely reported phenomenon. At this time, about 30 cases of pure, or *de novo*, MEC have so far been reported in the medical literature. While the histologic, immunohistochemical, and even ultrastructural features have been well described, a definite diagnosis of MEC based on cytology alone remains challenging. Herein, we report the case of a pure, infiltrating myoepithelial carcinoma of the breast while providing a correlation between the histologic and cytologic findings. We furthermore discuss the potential role of fine needle aspiration (FNA) within the diagnostic work-up of this neoplasm in relation to other spindle cell lesions of the breast.

## Case presentation

The patient was a 52-year-old white female who had noticed a gradually enlarging lump in her left breast for the past five years. On physical examination, the mass was well-circumscribed, non-tender to palpation, and freely mobile. Subsequent mammography revealed a complex 7-cm mass in the upper outer quadrant of the left breast. Fine needle aspiration, core needle biopsies, and finally a simple mastectomy with sentinel node biopsy were performed over the next several months. The patient's past medical and family histories included fibrocystic change of the breast, to which she attributed the lump.

### Cytologic findings

**Figure 1:**
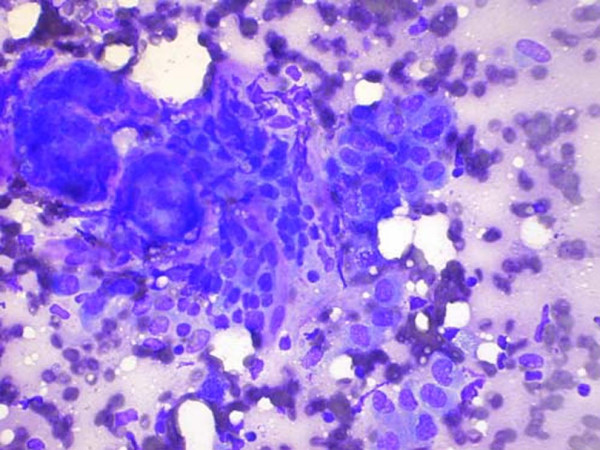
**(Diff-Quik stain, ×400)**: FNA smear from the breast mass showing a crowded, haphazardly arranged cluster of cells lying amidst a metachromatic stroma.

**Figure 2:**
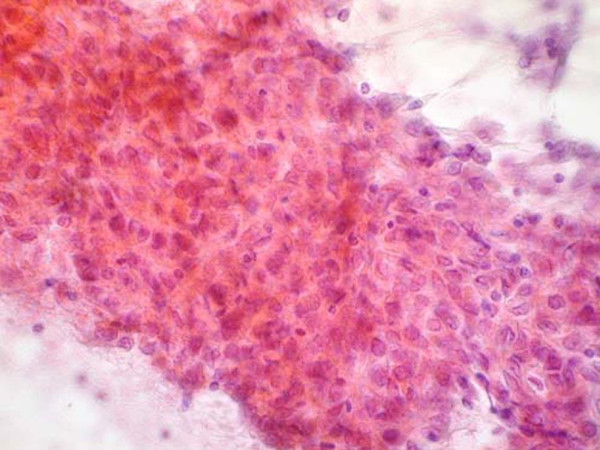
**(Papanicalaou stain, ×400)**: This nest of pleomorphic cells is composed of a mixture of large epithelioid, plasmacytoid, and spindle cells.

Three air-dried smears were stained with Diff-Quik while three alcohol-fixed smears were stained by the Papanicolaou method. Approximately 60 ml of cystic fluid drained from the same area of the breast was prepared as two smears, fixed with alcohol, and stained by Papanicolaou stain. These last two smears showed an abundance of macrophages and acute inflammatory exudate. The other six smears all showed high overall cellularity including multiple, scattered islands within a varying background of fat necrosis and a fibrillary, metachromatic stroma (Figure 1). These cell groups were composed of a haphazardly arranged mixture of large epithelioid, plasmacytoid cells with increased nuclear-to-cytoplasmic ratios (Figure 2). A distinct population of spindle cells with high nuclear-to-cytoplasmic ratios was also identified. The nuclei were finely vesicular with distinct nucleoli and showed considerable pleomorphism ranging from irregular, globoid forms to elongate, cigar-shaped objects. Several mitotic figures were identified, but necrosis was absent. The case was diagnosed as suspicious for malignancy, with metaplastic carcinoma of the breast as a differential diagnosis.

### Surgical pathology and immunohistochemistry

**Figure 3:**
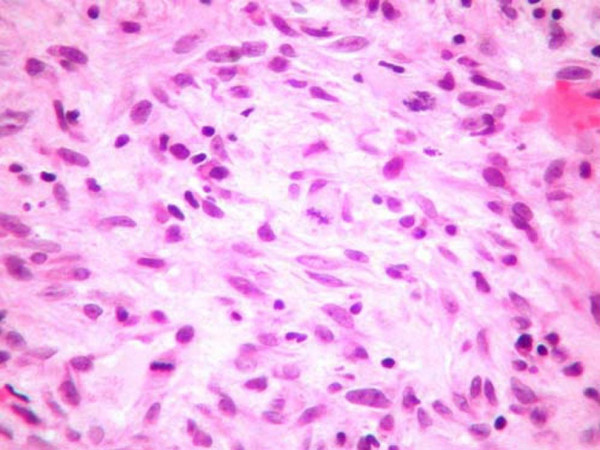
**(H&E, ×100)**: Histologic section revealing irregular dense proliferations of pleomorphic spindle cells. Mitoses were numerous.

**Figure 4:**
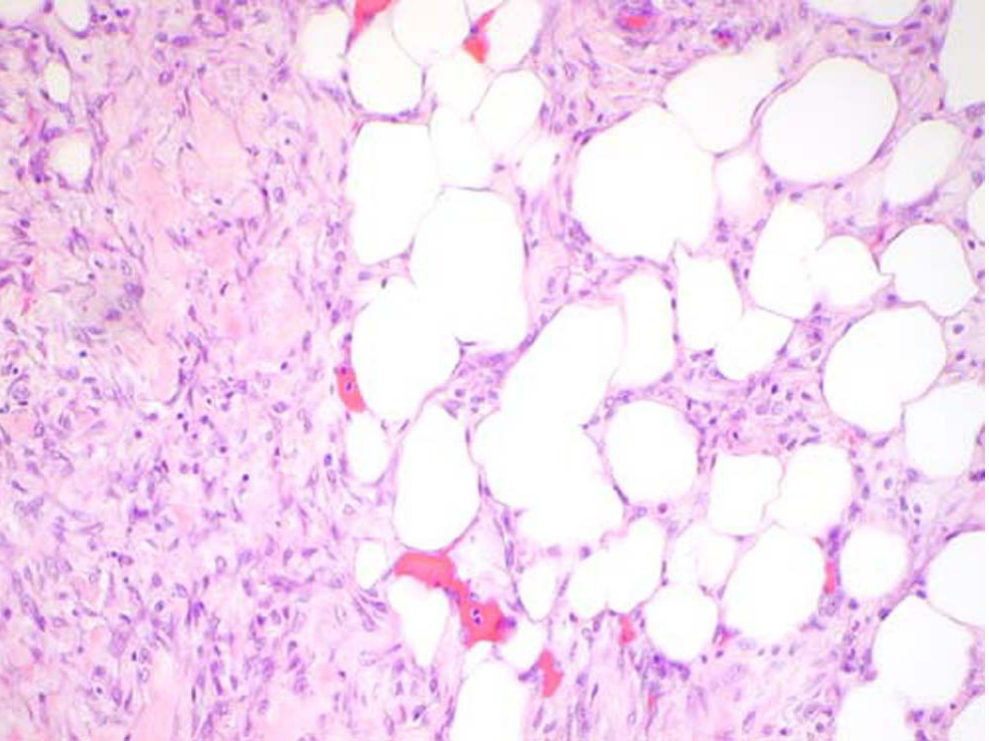
**(H&E, ×100)**: Histology from the edge of the tumor as the neoplastic cells invade the surrounding breast tissue (hematoxylin and eosin, ×100).

The mammotome biopsy samples were comprised of 19 fibrofatty tissue cores while the simple mastectomy specimen was a 21 × 17 × 5 cm portion of fibrofatty breast parenchyma with an overlying, unremarkable skin ellipse and areola-nipple complex. Serial sectioning of the mastectomy revealed a 6 × 4 × 4 cm biopsy cavity within the upper outer quadrant of the specimen. The periphery of the cavity had a firm, fibrous border and extended to within 0.7 cm of the deep surgical resection margin. The tissue from the biopsy cavity was sampled *en toto*. Microscopic examination of the core biopsy tissues and, subsequently, of the mastectomy specimen revealed irregular dense proliferations of plump spindle cells with moderate cytologic and nuclear pleomorphism (Figure 3). The majority of the nuclei showed prominent nucleoli. Mitotic figures were frequent (12 mitotic figures per 10 high power fields) and could be identified throughout the lesion. Additionally, tissue from the biopsy cavity demonstrated scattered, spotty foci of necrosis as well as both perivascular and perineural invasion. The edges of the lesion clearly infiltrated the surrounding breast parenchyma (Figure 4). All surgical resection margins were uninvolved by the neoplastic process. Other findings included atypical ductal hyperplasia, sclerosing adenosis, and periductal mastitis. The sentinel axillary lymph node was negative for malignancy.

**Figure 5:**
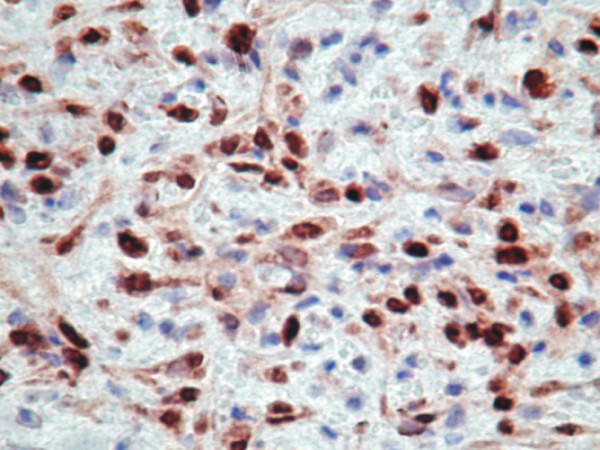
**(p63, ×100)**: The tumor cell nuclei stain strongly with the myoepithelial marker p63.

The proliferating spindle cells revealed diffuse positivity for the myoepithelial markers smooth muscle actin, CD10, and p63 (Figure 5) while showing focal positivity for S-100. Vimentin positivity was strong and diffuse. Additionally, increased positivity for Ki-67 provided evidence for a high proliferation index among the neoplastic cells. The immunohistochemical markers glial fibrillary acid protein (GFAP), desmin, estrogen receptor (ER), and progesterone receptor (PR) did not highlight cells from the lesion. Lastly, positive staining with the pancytokeratin marker [AE1/AE3 + 8/18] ruled out the possibility of a sarcoma with myoepithelial differentiation.

The histology and accompanying immunohistochemical staining patterns were consistent with an infiltrating myoepithelial carcinoma of the breast.

### Course

Approximately three months later the patient was seen at an outside institution, whereupon imaging studies revealed multiple bilateral lung lesions. A biopsy from the apex of the right lung and from the left lung confirmed pulmonary metastasis by the breast MEC. The patient died several months later.

## Discussion

The cytologic features of this lesion correlated well with the histologic findings. Architecturally, the hypercellularity and haphazard cellular arrangements were clearly demonstrated, as were several of the cellular findings including the spindle cell morphology, nuclear pleomorphism, conspicuous nucleoli, and the presence of mitoses. A feature that appeared more prominent on cytology, however, were the epithelioid and plasmacytoid morphologies of a large number of the tumor cells, whereas on histology the neoplastic cells were predominantly spindle-shaped, albeit plump.

There has been one report describing the cytologic findings of an intraductal mammary MEC [1]. The cytology, as in our case, showed cohesive cell groups composed of spindle cells with cigar-shaped nuclei showing atypia and mitoses. These spindle cells were also admixed with what they describe as "polygonal" cells, which appear quite similar to the epithelioid cells in our specimen. In contrast to our case, however, the authors report that the cells were organized in a fascicular pattern and that a subpopulation of cells with clear cytoplasm was present.

Furthermore, the cytologic features of a pure malignant MEC have recently been described by Sauer and are quite similar to our findings [2]. In contrast to our case, however, the malignant cells presented mostly as single cells rather than in clusters. Moreover, we did not appreciate the presence of occasional nuclear inclusions as did Sauer.

A wealth of different spindle cell lesions of the breast exist, translating into an accordingly wealthy number of differential diagnoses for a cytologic specimen displaying a predominant population of spindle cells. But, because of the multiple worrisome cytologic findings in this case, the list of likely diagnostic possibilities heavily favored malignant entities over benign processes. Such cytologic features have been discussed by Darvishian and Lin in which 17 myoepithelial cell-rich lesions, though mostly of salivary gland origin, showed that pleomorphism, coarse nuclear chromatin, prominent nucleoli, mitoses, and necrosis were observed only in the malignancies; 89% (8/9) of these malignant lesions were eventually diagnosed by histology as myoepithelial carcinoma [3].

Various mammary spindle cell malignancies typically show markedly pleomorphic histopathology and therefore may appear more or less indistinguishable from MEC on cytology. Metaplastic (sarcomatoid) carcinoma, spindle cell carcinoma, malignant fibrous histiocytoma, and other sarcomas all may demonstrate atypical spindle cells along with other common stigmata of cancer like necrosis and mitotic activity [4-6]. In some instances, the presence of such features as a chondromyxoid background and atypical multinucleated giant cells in a case of metaplastic carcinoma may generously help in eliminating MEC from the differential.

Myoepithelial cells may adopt a number of different morphologies on cytologic specimens including spindle cell, clear cell, epithelioid, and plasmacytoid forms [3]. In a study by Hornick and Fletcher, over half of their 101 cases of soft tissue myoepitheliomas actually showed a mixed pattern of these four morphologies [7]. Our case did not reveal a subpopulation of clear cells but did display the other three morphologies whereas the MEC reported by Sauer described both the spindle cell and epithelioid ("polygonal") cell populations [2]; the intraductal case reported by Tamai et al. lacked only the plasmacytoid cell type [1]. The heterogeneity with which myoepithelial cells may present on cytology can therefore complicate the diagnostic process for myoepithelial carcinoma even further, obviating a need to expand the differential diagnosis beyond spindle cell lesions to include biphasic entities such as metaplastic carcinoma and malignant phyllodes tumor [8].

In most cases of metaplastic carcinoma, the carcinomatous component is identified on cytology, allowing for a smoother diagnostic process. The problem arises when only the mesenchymal population is sampled. Strong keratin staining of the mesenchymal elements favors a metaplastic carcinoma over MEC, but differentiating between the two entities from a cytologic specimen may not always be possible.

It may be appropriate to entertain the diagnosis of lobular carcinoma or metastatic melanoma, simply because some of the tumor cells in our case displayed a plasmacytoid morphology. The presence of signet-ring cells in lobular carcinoma and intracytoplasmic pigment in melanoma can distinguish these entities from MEC [3].

Ultimately, in order to subclassify a spindle cell lesion like MEC based solely upon cytomorphology, immunohistochemistry will most likely be needed. By showing that the lesion is reactive for the immunohistochemical markers SMA, S-100, cytokeratin, p63, and CD10, a myoepithelial origin for the tumor can be established [3]. In our case, the immunohistochemical panel was performed on tissues derived from the mammotome biopsy, as the cytologic specimen was inadequate for staining procedures.

## Conclusion

What role, if any, does cytology play in diagnosing MEC of the breast? This case shows that an FNA specimen of a breast lesion showing a biphasic pattern with or without tumor necrosis or mitotic figures is adequate for assessing malignancy in a myoepithelial carcinoma. Besides MEC, the differential diagnosis for such findings includes metaplastic carcinoma and phyllodes tumor as two of the more likely candidates. However, arriving at a correct, specific diagnosis by cytology alone is not likely when one considers the substantial list of entities that may closely mimic this lesion on cytologic specimens. Here, the most important role of FNA is to answer, "Is this lesion a benign, or malignant, process?" This case revealed a number of different features such as nuclear pleomorphism, hypercellularity, and mitotic activity that allowed us to appreciate the neoplasm's malignant nature. Whether or not other MECs will consistently show such striking malignant features on cytology remains to be seen as more cases are reported.

## Competing interests

The author(s) declare that they have no competing interests.

## Authors' contributions

DL was the lead manuscript writer while YC and KK helped draft the manuscript and took part in the literature review with equal representation. KL revised the draft critically for intellectual content and guided the direction of the project. All authors read and approved the final manuscript.
